# CO Sensing Performance of a Micro Thermoelectric Gas Sensor with AuPtPd/SnO_2_ Catalyst and Effects of a Double Catalyst Structure with Pt/α-Al_2_O_3_

**DOI:** 10.3390/s151229873

**Published:** 2015-12-15

**Authors:** Tomoyo Goto, Toshio Itoh, Takafumi Akamatsu, Woosuck Shin

**Affiliations:** National Institute of Advanced Industrial Science and Technology (AIST), 2266-98 Shimo-Shidami, Moriyama-ku, Nagoya 463-8560, Japan; itoh-toshio@aist.go.jp (T.I.); t-akamatsu@aist.go.jp (T.A.)

**Keywords:** thermoelectric gas sensor, CO oxidation, noble metals, combustion catalyst, gas selectivity

## Abstract

The CO sensing properties of a micro thermoelectric gas sensor (micro-TGS) with a double AuPtPd/SnO_2_ and Pt/α-Al_2_O_3_ catalyst were investigated. While several nanometer sized Pt and Pd particles were uniformly dispersed on SnO_2_, the Au particles were aggregated as particles measuring >10 nm in diameter. *In situ* diffuse reflectance Fourier transform Infrared spectroscopy (DRIFT) analysis of the catalyst showed a CO adsorption peak on Pt and Pd, but no clear peak corresponding to the interaction between CO and Au was detected. Up to 200 °C, CO combustion was more temperature dependent than that of H_2_, while H_2_ combustion was activated by repeated exposure to H_2_ gas during the periodic gas test. Selective CO sensing of the micro-TGS against H_2_ was attempted using a double catalyst structure with 0.3–30 wt% Pt/α-Al_2_O_3_ as a counterpart combustion catalyst. The sensor output of the micro-TGS decreased with increasing Pt content in the Pt/α-Al_2_O_3_ catalyst, by cancelling out the combustion heat from the AuPtPd/SnO_2_ catalyst. In addition, the AuPtPd/SnO_2_ and 0.3 wt% Pt/α-Al_2_O_3_ double catalyst sensor showed good and selective CO detection. We therefore demonstrated that our micro-TGS with double catalyst structure is useful for controlling the gas selectivity of CO against H_2_.

## 1. Introduction

Breath gas contains a range of marker gases that can be associated with disease and metabolism [1,2]. These gases, including CO, H_2_, and CH_4_, are present in concentrations of several ppm, and hence, accurate analysis of low concentrations of the gases is important for medical applications. In addition, for regular hospital health checks, the gas detector must be easy to adjust and give a fast response. We propose a micro thermoelectric gas sensor (micro-TGS), which uses the thermoelectric detection of a combustion catalyst, for monitoring H_2_ [3,4], CO [5,6,7], and CH_4_ [8,9] in human breath gas. The micro-TGS is an inflammable gas sensor with promising gas responsivity, and shows a clear linear relationship to allow the detection of gases at the ppm level [3].

Au-loaded catalysts have been reported for use in micro-TGSs for CO detection, with Au/Co_3_O_4_ catalysts exhibiting high selectivity to CO but low detection sensitivity [5,6]. Loading of Au, Pt, and Pd on cobalt oxide (Co_3_O_4_ and CoO) catalysts has been reported to improve the detection sensitivity to CO [7], as Pt and Pd particles are also widely used as catalysts for CO oxidation [10,11]. The micro-TGS containing the AuPtPd/CoO catalyst exhibited improved performance at 1 ppm CO in dry air, and good selectivity towards CO over H_2_ gas [7]. However, a reduction in CO sensitivity of the cobalt oxide catalysts was observed upon continuous use of the micro-TGS [6]. It is assumed that the deactivation of Au particles [12] or cobalt oxide [13] may cause this reduction in CO sensitivity. We therefore chose to focus on SnO_2_ as a catalyst support for the micro-TGS. SnO_2_ is a well-known n-type semiconducting oxide, and is used in semiconductor-type gas sensors. In addition, studies into the CO oxidation activity of SnO_2_ catalysts either with or without noble metals have been previously reported [14,15,16]. Sakai and Itoh reported the sensing properties of Pt, Pd, and Au loaded on SnO_2_ (AuPtPd/SnO_2_) thick films as semiconductor-type gas sensors for volatile organic compounds (VOCs). As the addition of three noble metals to SnO_2_ appears to improve their sensitivity to VOCs [17,18], we attempted the incorporation of an AuPtPd/SnO_2_ combustion catalyst in our micro-TGS for CO detection.

In the micro-TGS systems, the selectivity of the AuPtPd/SnO_2_ catalyst must be easily controllable. The H_2_ selectivity for the micro-TGS containing a Pt/α-Al_2_O_3_ catalyst can be controlled by the operating temperature, as the catalyst operating temperature is approximately 100 °C [3,4]. However, for CO and CH_4_, selectivity is lower, as a higher catalyst temperature is required for the oxidation of CO and CH_4_. In this context, we previously attempted to control CO and CH_4_ selectivity using a “double catalyst structure” [8,19]. This structure adjusts the balance of the combustion heats of catalysts deposited on the thermoelectric film in the micro-TGS device. Thus, changes in the sensing properties of the micro-TGS are expected when employing the double catalyst structure.

We herein report our investigations into the sensing properties of an AuPtPd/SnO_2_ catalyst on a micro-TGS using a double catalyst structure design. The CO oxidation properties and sensing properties were compared using diffuse reflectance infrared Fourier transform spectroscopy and morphological observations. Furthermore, the effects of the double catalyst structure containing a Pt/α-Al_2_O_3_ catalyst on the selective CO sensing properties of the micro-TGS are also discussed.

## 2. Experimental Section

### 2.1. Preparation of the Double Catalyst Micro-TGS

The micro-TGS device measuring 4 × 4 mm^2^ was composed of a p-type B-doped SiGe pattern, a Pt heater, and an electrode line pattern on a double-sided polished Si substrate. Processing details for the B-doped SiGe thin film and its pattern were reported previously [4]. The Pt micro-heater and electrode lines were prepared using a lift-off technique, and the reverse side of the Si substrate was etched out using an aqueous KOH solution to prepare the membrane structure.

**Figure 1:**
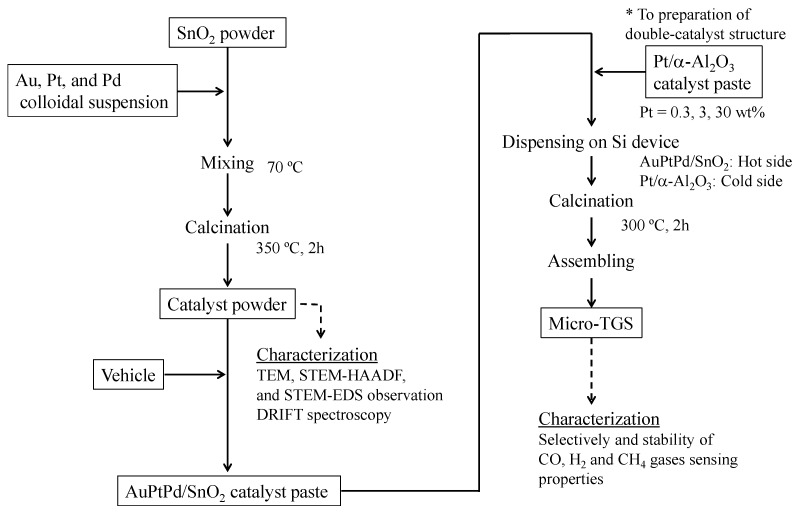
Schematic representation of the AuPtPd/SnO_2_ catalyst powder and micro thermoelectric gas sensor (micro-TGS) preparation.

The AuPtPd/SnO_2_ catalyst powder was prepared by reference to Itoh’s method [18] and is represented schematically in Figure 1. Au, Pt, and Pd colloids were purchased from Tanaka Kikinzoku Kogyo K.K., Tokyo, Japan, respectively. A catalyst containing 1 wt% Au (2 wt% Au colloidal suspension), 1 wt% Pt (4 wt% Pt colloidal suspension), and 1 wt% Pd (4 wt% Pd colloidal suspension) was added to SnO_2_ powder (particle size 100 nm; Sigma-Aldrich, St. Louis, MO, USA) in water. Mixing was carried out at 70 °C, followed by drying at 90 °C to give the catalyst powder. This powder was calcined at 350 °C in an electric furnace for 2 h under air. The calcined powder was then dispersed in ethanol, and the floating particles retrieved and mixed with a vehicle containing terpineol, ethyl cellulose, and distilled water (9:1:5 wt. ratio) to give a paste. The catalyst pastes were then applied to the micro-TGS device using an air dispenser (MUSASHI Engineering, Inc., Tokyo, Japan), and the devices were calcined at 300 °C for 2 h. Finally, the devices were mounted on stems, and connected using gold-bonding wire to prepare the micro-TGS.

To enhance the micro-TGS selectivity of CO over H_2_, a double catalyst structure was employed, as described in previous reports [8]. The Pt/α-Al_2_O_3_ catalyst powder (0.3, 3, and 30 wt%) for H_2_ combustion was prepared by mixing and subsequent calcination at 300 °C, as described previously [3,4].

**Figure 2:**
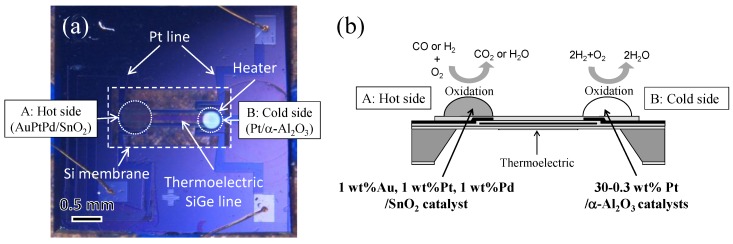
Optical images of the AuPtPd/SnO_2_ and Pt/Al_2_O_3_ catalysts on the Si membrane of the micro-TGS (**a**), and a side view of the double catalyst structure (**b**).

**Figure 3:**
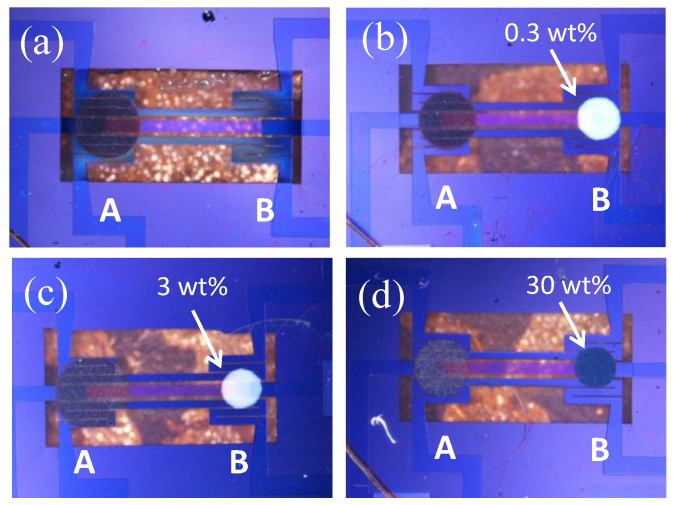
Double catalyst structures with AuPtPd/SnO_2_ (A: Hot side) and 0.3–30 wt% Pt/α-Al_2_O_3_ (B: Cold side) catalysts for the micro-TGS: (**a**) No Pt catalyst; (**b**) 0.3 wt% Pt catalyst; (**c**) 3 wt% Pt catalyst, and (**d**) 30 wt% Pt catalyst.

The structure and working principle of the double catalyst micro-TGS are based on our previously reported study [8,19]. As shown in Figure 2, AuPtPd/SnO_2_ and 0.3–30 wt% Pt/α-Al_2_O_3_ catalysts were deposited on the hot (point A) and cold (point B) side of a micro-TGS device, respectively. Hot side indicates a position to commonly put a catalyst, and cold side show the position of without catalyst on micro-TGS. It is expected that the AuPtPd/SnO_2_ catalyst will oxidize both H_2_ and CO. However, Pt/α-Al_2_O_3_ is a combustion catalyst for H_2_, [3,4] hence, the combustion heat of the Pt/α-Al_2_O_3_ catalyst (at point B) reduces the temperature difference between the points A and B. As a result, when a mixture of H_2_ and CO is introduced into the calorimetric-TGS device, the sensor response of the AuPtPd/SnO_2_ catalyst to H_2_ will be inhibited. The design and composition of single and double catalyst type structures of the AuPtPd/SnO_2_ and 0.3–30 wt% Pt/α-Al_2_O_3_ catalysts of the micro-TGSs are given in Figure 3 and Table 1.

**Table 1:** Design of AuPtPd/SnO_2_ and Pt/α-Al_2_O_3_ double catalyst structures.

Notation	Design	Pt content in Pt/α-Al_2_O_3_
		wt%
0Pt	AuPtPd/SnO_2_ + no catalyst	0
0.3Pt	AuPtPd/SnO_2_ + Pt/α-Al_2_O_3_	0.3
3Pt	AuPtPd/SnO_2_ + Pt/α-Al_2_O_3_	3
30Pt	AuPtPd/SnO_2_ + Pt/α-Al_2_O_3_	30

### 2.2. Catalyst Powder Characterization

The morphology of the catalyst powder was observed by transmission electron microscopy (TEM; Tecnai Osiris, FEI, OR, USA) with high-angle annular dark field scanning transmission electron microscopy (STEM-HAADF), at an accelerating voltage of 200 kV. Elemental mapping of Au, Pt, Pd, and Sn was carried out using STEM-energy dispersive X-ray spectroscopy (STEM-EDS). CO adsorption and oxidation of the catalyst powder were investigated by diffuse reflectance Fourier transform Infrared spectroscopy (DRIFT) analysis (Nexus 470 FTIR, Nicolet, Waltham, MA, USA), at 25, 50, 100, 200, and 300 °C. As measurement gases, 10,000 ppm CO in dry air and 10,000 ppm CO in Ar were used at a flow rate of 50 cm^3^/min. DRIFT spectra were measured in absorbance mode without switching carrier gas (Air or Ar).

### 2.3. Investigation of Gas Sensing Properties

**Figure 4:**
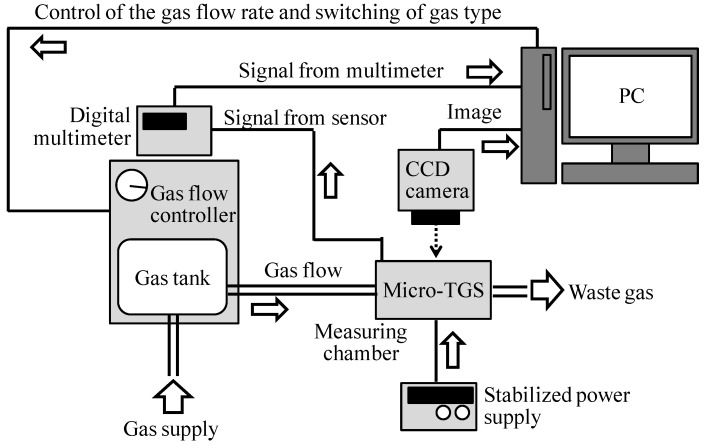
Measurement system of gas sensing properties of micro-TGS.

Measurement system of sensing properties of micro-TGS is shown in Figure 4. The sensor response of micro-TGS for H_2_, CO, and CH_4_ was investigated using a gas flow chamber with a volume of 60 cm^3^. The voltage applied to the micro-TGS device was adjusted to control the heater temperatures (50–250 °C) and was monitored using an IR camera (LAIRD-270A, Nikon Co., Tokyo, Japan). To test the micro-TGS, pure dry air was allowed to flow into the chamber together with the test gases (in dry air) in the following order: Dry air, test gas, and dry air. Each step lasted 90 s with a gas flow rate of 200 cm^3^/min. The voltage across the thermoelectric thin film of the micro-TGS varied by the combustion heat of the catalysts, a phenomenon based on the Seebeck principle. The voltage signal (Vs) was corrected using a digital multimeter (K2700, TFF Co. Keithley Inst., Tokyo, Japan) following pretreatment of the double catalyst micro-TGS with 10,000 ppm H_2_ in dry air at 200 °C. The response of Vs (ΔV) is defined by Equation (1):

*ΔV* = *Vs*_(170–180)_ – *Vs*_(80–90)_ (1)

where *Vs*_(170–180)_ and *Vs*_(80–90)_ are the averages of Vs over 170–180 s (in test gas) and 80–90 s (in dry air), respectively. The sensor response was investigated by measuring the voltage across the micro-TGS at 1000 ppm CO, H_2_, and CH_4_ at 50–250 °C and 10–10,000 ppm CO or H_2_ at 200 °C. The stability of the sensor responses was determined by subjecting the micro-TGS to a gas sensitivity test at 200 °C, for 1, 2, and 9 days.

## 3. Results and Discussion

### 3.1. Catalyst Microstructure

**Figure 5:**
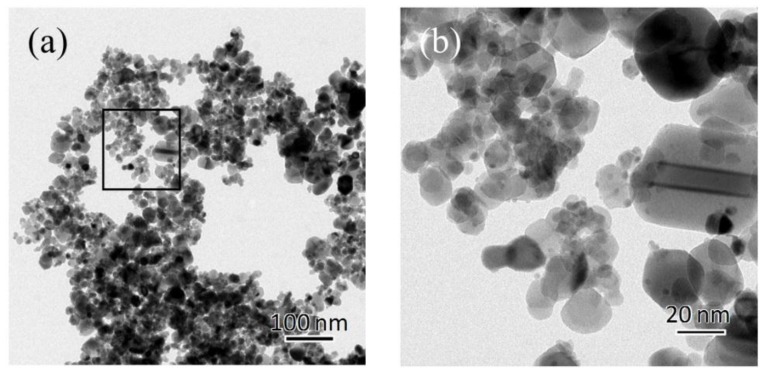
Transmission electron microscopy (TEM) images of the AuPtPd/SnO_2_ catalyst powder: (**a**) low magnification (17,500) and (**b**) high magnification (88,000).

Figure 5 shows the TEM images of the AuPtPd/SnO_2_ catalyst powder. Both well-dispersed SnO_2_ particles (20–50 nm) (Figure 5a), and small metal particles (3 nm) supported on SnO_2_ particles can clearly be seen (Figure 5b). It should be noted here that the high combustion performance of the catalyst is affected by its porous structure and particle size. The grain size of the aggregated metal particles was several nanometers, similar to the starting colloidal particle, resulting in relatively good dispersion. However, to investigate whether each metal element (*i.e.*, Pt, Pd, and Au) is alloyed or not, the catalyst was analyzed by STEM and elemental mapping.

**Figure 6:**
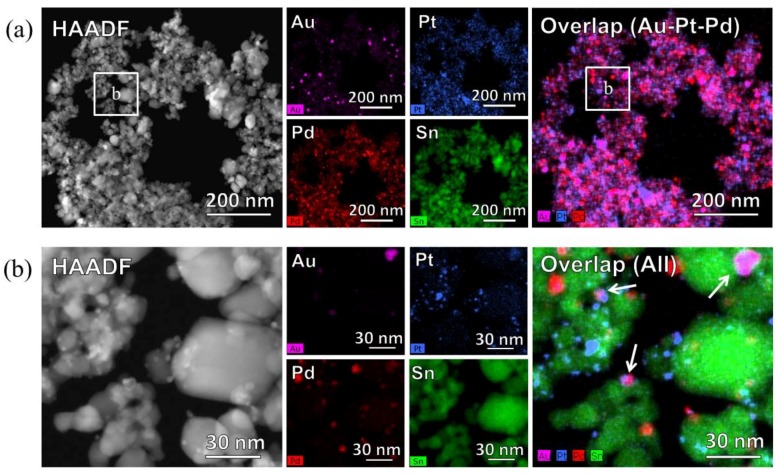
High-angle annular dark field scanning transmission electron microscopy (STEM-HAADF) and STEM-energy dispersive X-ray spectroscopy (STEM-EDS) images of the AuPtPd/SnO_2_ catalyst powder. (**a**) STEM-HAADF image and EDS maps of Au, Pt, Pd, Sn, and Overlap (Au-Pt-Pd) at low magnification (110,000), and (**b**) STEM-HAADF image and EDS maps of Au, Pt, Pd, Sn, and Overlap at high magnification (630,000).

Figure 6 shows the High-angle annular dark field scanning transmission electron microscopy (STEM-HAADF) images and STEM-energy dispersive X-ray spectroscopy (STEM-EDS) mapping of the Au, Pt, Pd, and Sn distributions in the catalyst powder. Low magnification images (Figure 6a) indicate that the noble metals were dispersed over the SnO_2_ particles. However, in the high magnification images (Figure 6b), only the Pt and Pd nanoparticles were deposited and dispersed on SnO_2_ particles, while the Au nanoparticles aggregated as 10 nm particles, overlapping with Pt and Pd, as indicated by arrows in the overlap image of Figure 6b.

### 3.2. DRIFT Characterization of the Catalyst

**Figure 7:**
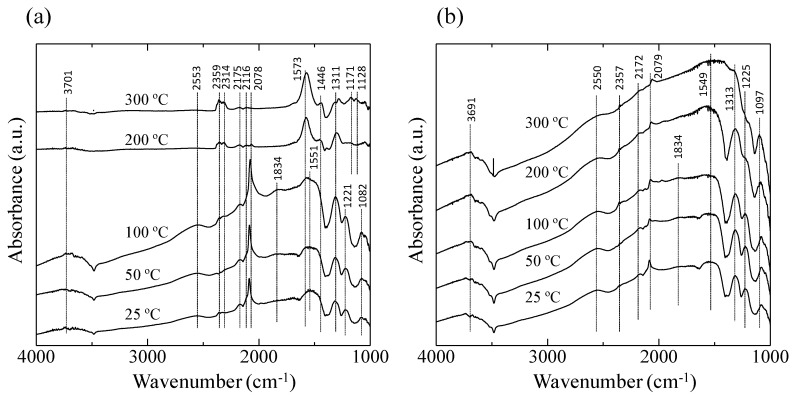
Diffuse reflectance Fourier transform Infrared spectroscopy (DRIFT) spectra of the AuPtPd/SnO_2_ catalyst powder at 25–300 °C, in (**a**) 10,000 ppm CO in dry air, and (**b**) 10,000 ppm CO in Ar.

Figure 7 shows the 1000–4000 cm^−1^ region of the diffuse reflectance Fourier transform Infrared spectroscopy (DRIFT) spectra of the AuPtPd/SnO_2_ catalyst at 25–300 °C in 10,000 ppm CO in both dry air (Figure 7a) and in Ar (Figure 7b). As can be seen in Figure 7a, at lower temperatures (25–100 °C), bands at 1082, 1221, 1311, and 1551 cm^−1^ were observed, corresponding to vibrations of the carbonate species on noble metals or SnO_2_ [15,20]. The bands at 1834, 2078, 2116, and 2175 cm^−1^ could be assigned to CO. In particular, the sharp peak at 2078 cm^−1^ and the broad peak at 1834 cm^−1^ correspond to the linear and bridging bonds of the CO-Pt and CO-Pd bands, respectively [21]. The peak intensity corresponding to CO adsorption decreased with increasing the catalyst temperature. At catalyst temperatures of 200 and 300 °C, bands at 2314 and 2359 cm^−1^, corresponding to CO_2_, were observed, confirming the oxidation of CO by the AuPtPd/SnO_2_ catalyst. For the experiments employing 10,000 ppm CO in Ar (Figure 7b), the peaks corresponding to the carbonate species (at 1097, 1225, 1313, 1549, 2079, and 2172 cm^−1^) were detected at 25–200 °C. However, in contrast to the experiments employing 10,000 ppm CO in dry air (Figure 7a), peaks corresponding to CO_2_ were not detected.

The DRIFT spectra (Figure 7) clearly showed a peak corresponding to CO adsorption on the Pt and Pd particles at 2078 cm^−1^, but no sharp peak corresponding to CO adsorption on the metallic Au site was detected at 2112 cm^−1^ as shown in previous researches [15,22]. This indicated that less CO oxidation took place on the Au particles compared to the Pt and Pd particles. As shown in the STEM-EDS images (Figure 6), Au particles formed aggregates, thus increasing their particle size (≥10 nm). This can be explained by previous studies showing that the oxidation reaction of the Au catalyst slowed with increasing particle diameter, with a particle size of <10 nm being required [12]. In addition, Haruta *et al.*, reported that the combination of Au with transition metal oxides resulted in high catalytic activities [23]. Therefore, CO oxidation by Au would be expected to be lower than that of Pt or Pd when considering the large particle size and the effect of non-transition metal (SnO_2_) catalysis carriers.

### 3.3. Sensor Gas Response

**Figure 8:**
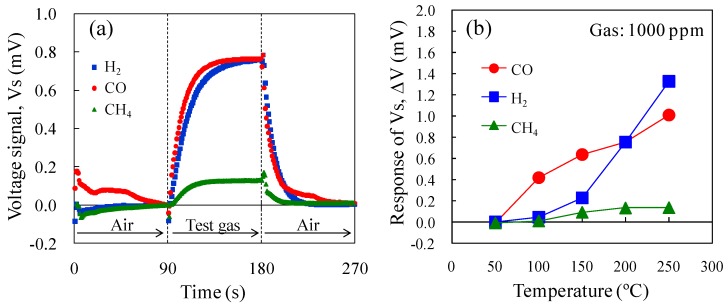
Vs of the AuPtPd/SnO_2_ micro-TGS for 1000 ppm H_2_, CO, and CH_4_ in dry air. (**a**) Response curves of the micro-TGS at a catalyst temperature of 200 °C, and (**b**) the ΔV of the micro-TGS at 25–300 °C.

Figure 8a shows the Vs of the micro-TGS with AuPtPd/SnO_2_ catalyst at 200 °C for 1000 ppm CO, H_2_, and CH_4_. The combustion performance of the catalyst is directly related to the Vs of the sensor. At a catalyst temperature of 200 °C, the CO and H_2_ sensing properties of the micro-TGS were comparable, while CH_4_ combustion was not effective and the Vs was low. The temperature dependence of the ΔV of the AuPtPd/SnO_2_ micro-TGS for 1000 ppm CO, H_2_, and CH_4_ is shown in Figure 8b. The H_2_ and CO sensing signals increased to 1.4 and 1.0 mV at 250 °C, respectively. Up to temperatures of 200 °C, the Vs for CO of this micro-TGS was higher than that of ΔV for H_2_. In addition, the CH_4_ sensing signal increased slightly to 0.14 mV at 250 °C. Considering our previously reported results [9] on a micro-TGS with Pd/θ-Al_2_O_3_ catalyst, to achieve ppm detection of CH_4_, a catalyst temperature of 400 °C was required. Thus, the catalyst temperature of 250 °C used in the present study was too low to achieve CH_4_ combustion.

**Figure 9:**
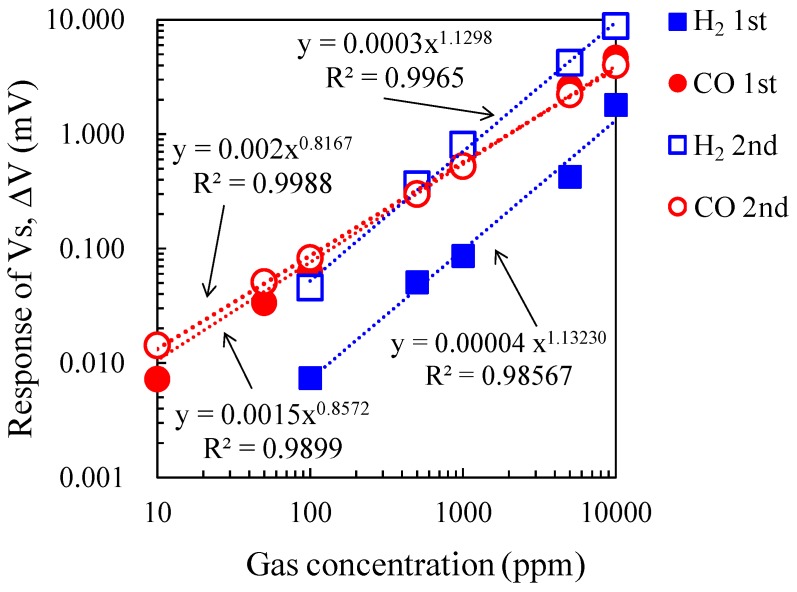
ΔV against CO and H_2_ concentration for the micro-TGS, from 10–10,000 ppm in dry air.

**Figure 10:**
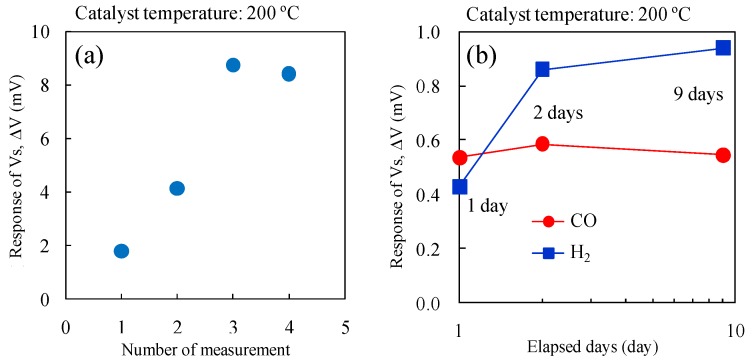
Gas response of the micro-TGS with AuPtPd/SnO_2_ catalyst: (**a**) ΔV against time for the micro-TGS with 10,000 ppm H_2_ in dry air; and (**b**) long-term stability of ΔV from the micro-TGS with AuPtPd/SnO_2_ catalyst for 1000 ppm H_2_ in dry air or 1000 ppm CO in dry air at 200 °C for 1–9 days.

Figure 9 shows the double logarithmic plot of gas concentration and ΔV of the micro-TGS with AuPtPd/SnO_2_ catalyst at 200 °C for 10–10,000 ppm of CO and H_2_. The plots were fitted to the power approximation curve “*y = ax^b^*”, and the sensor ΔV for these gases showed good linear dependence. While the gas concentration dependence of ΔV for CO was unchanged, the ΔV of the second measurement for H_2_ increased linearly, and the values became comparable to those of the ΔV for CO. Figure 10a shows the change in ΔV for the micro-TGS with AuPtPd/SnO_2_ during repeated measurements with 10,000 ppm H_2_ at a catalyst temperature of 200 °C. The long-term stability of the ΔV of the micro-TGS at 1000 ppm CO or H_2_ in dry air at 200 °C is shown in Figure 10b. The CO response of the micro-TGS remained fairly constant, while the H_2_ response increased with time. It therefore appears that the chemical state, composition, or dispersed state of Pt and Pd nanoparticles on SnO_2_ was altered by pretreatment with 10,000 ppm H_2_ in dry air.

We previously reported the CO response of the micro-TGS with noble metal-loaded cobalt oxide catalysts [5,6,7]. The ΔV of the AuPtPd/SnO_2_ micro-TGS for CO was comparable to that of the micro-TGS with either AuPtPd/Co_3_O_4_ or AuPtPd/CoO catalysts [7]. In addition, the stability of ΔV of the micro-TGS with AuPtPd/SnO_2_ catalyst observed in this study was higher than that of the previously reported micro-TGS with Au/Co_3_O_4_ catalyst [6]. These results indicate that the AuPtPd/SnO_2_ micro-TGS is useful as a CO gas sensor. Furthermore, this study revealed that this micro-TGS is required to control the varying ΔV of H_2_ in order to improve its CO selectivity against H_2_.

### 3.4. Double Catalyst Structure

We previously attempted implementing the double catalyst micro-TGS structure for use as a hydrogen sensor [19], along with calorimetric-TGS devices [8] to control sensor selectivity. In the present study, to enhance CO selectivity, changes in the CO and H_2_ sensing properties of the micro-TGS with AuPtPd/SnO_2_ catalyst containing a double catalyst structure were investigated. The AuPtPd/SnO_2_ catalyst, which was deposited on the hot side (point A) of the micro-TGS device, burned both H_2_ and CO gases. In contrast, the Pt/α-Al_2_O_3_ catalyst, which was deposited on the cold side (point B) of the micro-TGS device, oxidized only H_2_. We therefore aimed for the combustion heat of H_2_ (Q_H2_) to deduct from the combustion heat of H_2_ and CO (Q_H2_ + Q_CO_) for the AuPtPd/SnO_2_ catalyst, by employing various Pt contents for the Pt/α-Al_2_O_3_ catalyst.

**Figure 11:**
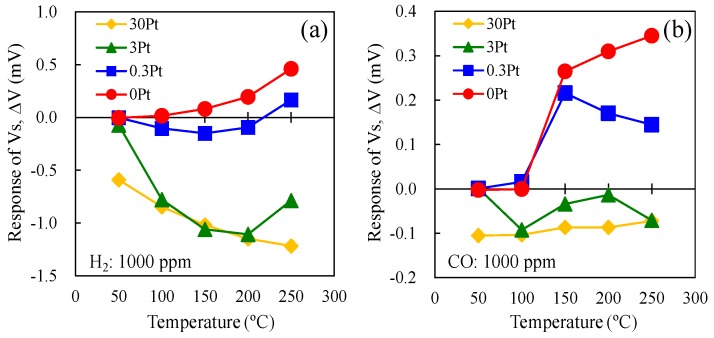
The ΔV of the single and double catalyst structure micro-TGS with AuPtPd/SnO_2_ and 0.3–30 wt% Pt/α-Al_2_O_3_ catalyst at 200 °C for (**a**) 1000 ppm H_2_, and (**b**) 1000 ppm CO in dry air.

Figure 11 shows the temperature dependence of the ΔV of the double catalyst micro-TGS with 1000 ppm H_2_ in dry air and 1000 ppm CO in dry air. The “0Pt” indicated the different batch of the same composition of AuPtPd/SnO_2_ micro-TGS. For the H_2_ gas response (Figure 11a), the values of ΔV for 3Pt and 30Pt were negative at all catalyst temperature, indicating a higher Q_H2_ for Pt/α-Al_2_O_3_ than for AuPtPd/SnO_2_. The ΔV of 0.3Pt was negative at all catalytic temperatures except 250 °C. Thus, the amount of Pt in the Pt/α-Al_2_O_3_ catalyst greatly influenced the H_2_ sensitivity of the AuPtPd/SnO_2_ micro-TGS, which is in agreement with previously reported calorimetric-TGS devices [8]. In addition, the ΔV of 0Pt (in the absence of Pt/α-Al_2_O_3_ catalyst on the cold side) was positive, and increased with increasing catalyst temperature. For the CO response (Figure 11b), the ΔV values of 3Pt and 30Pt were also negative between 50–250 °C, indicating that the Q_CO_ from Pt/α-Al_2_O_3_ containing high quantities of Pt is significant in the double catalyst micro-TGS system. Furthermore, ΔV of 0.3Pt increased upon increasing the catalyst temperature to 150 °C, and then decreased gradually at higher temperatures. Figure 11a,b indicates that the Pt/α-Al_2_O_3_ catalyst oxidized both H_2_ and CO, and that the combustion heats of both 30Pt and 3Pt were higher than that of the AuPtPd/SnO_2_ catalyst, due to a negative ΔV. The high Pt content in the Pt/α-Al_2_O_3_ catalyst therefore appeared to accelerate the oxidation reaction of H_2_ and CO. Indeed, this Pt/α-Al_2_O_3_ catalyst is a combustion catalyst for various gases, including both H_2_ and CO. With an increase in Pt content on the cold (point B) side of the Pt/α-Al_2_O_3_ catalyst, a decrease in the ΔV of H_2_ and CO was observed for the micro-TGS. Thus, ΔV varied in the order: 0Pt > 0.3Pt ~ 3Pt > 30Pt. Considering these results, an optimal Pt content of <3 wt% should be used in the Pt/α-Al_2_O_3_ catalyst to give the best CO selectivity.

**Figure 12:**
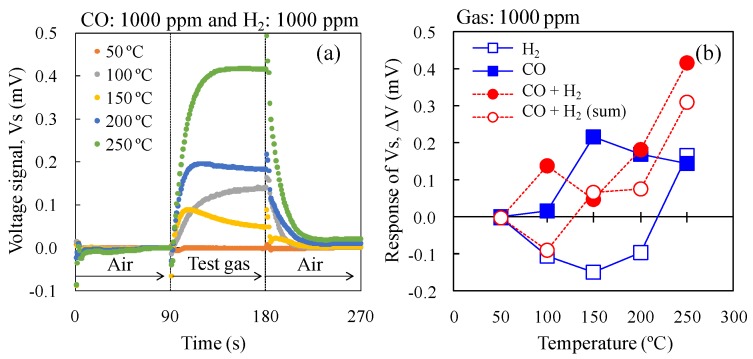
Vs of the double catalyst micro-TGS with AuPtPd/SnO_2_ and 0.3 wt% Pt/α-Al_2_O_3_ catalysts. (**a**) Response curves of the micro-TGS at catalyst temperatures of 25–300 °C for mixed gas (1000 ppm CO and 1000 ppm H_2_) in dry air; (**b**) Catalyst temperature dependence of the ΔV of the micro-TGS for 1000 ppm CO, 1000 ppm H_2_, mixed gas (1000 ppm CO + 1000 ppm H_2_), and calculated data (the sum of single gas data).

To investigate the CO selectively against H_2_, the ΔV of 0.3Pt to CO and H_2_ mixed gas in dry air was investigated (Figure 12), with the ΔV of 0.3Pt to CO and H_2_ shown in Figure 11 re-plotted in Figure 12b. At a catalyst temperature of 100 °C, the ΔV of 0.3Pt to the mixed gas was large, whereas those to CO and H_2_ were small and negative, respectively. It appears that the presence of H_2_ accelerated the oxidation of CO in the mixed gas on the AuPtPd/SnO_2_ catalyst. The combustion heat of the 0.3 wt% Pt/α-Al_2_O_3_ catalyst increased at 150 °C, due to the gradual decrease in ΔV with increasing temperature. In addition, between 150–250 °C, the ΔV of CO and H_2_ mixed gases increased with increasing catalyst temperature. At a catalyst temperature of 200 °C, the ΔV of 0.3Pt for the mixed gases was comparable to that for CO, indicating that 0.3Pt shows good CO selectivity against H_2_. Furthermore, at 250 °C, the ΔV of 0.3Pt to the mixed gas reached its highest value. However, the ΔV of 0.3Pt to H_2_ was positive while that to the mixed gas was comparable to the sum of the values for CO and H_2_. This indicates that the selectivity to CO of 0.3Pt decreased at a catalyst temperature of 250 °C. We therefore concluded that 0.3Pt gave the best CO selectivity against H_2_ on the double catalyst micro-TGS system at 200 °C.

From Figure 12b, the ΔV of experimental data of mixed gas was higher than that of calculated data (the sum of single gas data), at catalyst temperature of 100 °C. This result indicated that, at 100 °C, the combustion of 0.3 wt% Pt/α-Al_2_O_3_ for CO and H_2_ (cold side) was inhibited by adsorption of CO, compared to the case of AuPtPd/SnO_2_ catalyst (Hot side). On the other hand, the CO combustion on the AuPtPd/SnO_2_ catalyst (hot side) was relatively not inhibited much. Therefore combustion heat is generated and ΔV of experimental data showed the positive value. However the ΔV of experimental data was similar to that of calculated data from 150 °C and more. Therefore, at catalyst temperature of 200 °C, the tendency of ΔV of mixed gas could be estimated from the sum of ΔV of pure H_2_ and pure CO.

## 4. Conclusions

We investigated the CO, H_2_, and CH_4_ sensing properties of a micro thermoelectric gas sensor (micro-TGS) containing the AuPtPd/SnO_2_ catalyst as a CO gas sensor. CO oxidation by the catalyst powder was investigated, with the catalytic oxidation of CO by Pt and Pd particles being prominent compared to that by Au particles. This performance was related to the difference in size and dispersion of nanoparticles. While several nanometer sized Pt and Pd particles were uniformly dispersed on SnO_2_, the Au particles aggregated to form particles of >10 nm diameter. *In situ* DRIFT analysis of the catalyst showed a CO adsorption peak on Pt and Pd, but no clear interaction peak between CO and Au was detected. CO combustion by the AuPtPd/SnO_2_ catalyst was more temperature dependent than that of H_2_ up to 200 °C, with H_2_ combustion being activated by repeated exposure of H_2_ gas during the periodic gas test. Selective CO sensing against H_2_ of the micro-TGS was then attempted using a double catalyst structure containing 0.3–30 wt% Pt/α-Al_2_O_3_ as a counterpart combustion catalyst. The CO and H_2_ output voltage signal of the double catalyst micro-TGS decreased with an increase in Pt content in the counterpart catalyst, with optimal selective CO detection being achieved with the micro-TGS containing 0.3 wt% Pt/α-Al_2_O_3_. Future studies are now planned to investigate the gas concentration dependence of the double catalyst micro-TGS response voltage for breath analysis.
